# Factors Associated with Health-Related Quality of Life in Patients with Benign Prostatic Hyperplasia Undergoing Pharmacological Treatment: A Cross-Sectional Study

**DOI:** 10.3390/medicina61122244

**Published:** 2025-12-18

**Authors:** Srđan Govedarica, Aleksandar Rašković, Saša Vojinov, Dragan Grbić, Mladen Popov, Biljana Vučković, Dragan Zečević, David Strilić, Dimitrije Jeremić

**Affiliations:** 1 Clinic of Urology, University Clinical Center of Vojvodina, Hajduk Veljkova 1-9, 21000 Novi Sad, Serbia; sasa.vojinov@mf.uns.ac.rs (S.V.); d_grbic@yahoo.com (D.G.); mladen.popov@mf.uns.ac.rs (M.P.); dimitrije.jeremic@mf.uns.ac.rs (D.J.); 2 Faculty of Medicine, University of Novi Sad, Hajduk Veljkova 3, 21000 Novi Sad, Serbia; 902027d24@mf.uns.ac.rs; 3 Department of Pharmacology and Toxicology, Faculty of Medicine, University of Novi Sad, Hajduk Veljkova 3, 21000 Novi Sad, Serbia; aleksandar.raskovic@mf.uns.ac.rs; 4 Department of Pathophysiology and Laboratory Medicine, Faculty of Medicine, University of Novi Sad, Hajduk Veljkova 3, 21000 Novi Sad, Serbia; biljana.vuckovic@mf.uns.ac.rs; 5 International Center for Cardiovascular Diseases, MC Medicor, Polje 40, 6310 Izola, Slovenia; zeka64@mts.com

**Keywords:** benign prostatic hyperplasia, lower urinary tract symptoms, international prostate symptom score, health-related quality of life, whoqol-bref, pharmacological treatment

## Abstract

*Background and Objectives*: Benign prostatic hyperplasia (BPH) and associated lower urinary tract symptoms (LUTSs) can substantially impair health-related quality of life (HRQoL). We examined the relationship between LUTS severity, measured by the International Prostate Symptom Score (IPSS), and HRQoL assessed with the World Health Organization Quality of Life-BREF (WHOQOL-BREF) in men receiving pharmacological treatment for BPH. *Materials and Methods*: We conducted a cross-sectional study at the University Clinical Center of Vojvodina (May–July 2024). Seventy men aged 50–80 years on ≥1 year of pharmacological therapy for BPH were enrolled. LUTS severity was categorized by IPSS (mild, moderate, severe). HRQoL was measured across WHOQOL-BREF domains (physical, psychological, social, environmental). Group differences were tested with one-way analysis of variance (ANOVA) and post hoc tests; associations were evaluated with Spearman’s rank correlation; multivariable linear regression adjusted for age, socioeconomic status, and therapy type. *Results*: Severe LUTS were associated with significantly lower HRQoL in the physical (*p* = 0.002), social (*p* = 0.007), and environmental (*p* = 0.008) domains compared with mild or moderate symptoms, while psychological scores did not differ. IPSS correlated negatively with the physical (ρ = −0.438, *p* < 0.001), social (ρ = −0.470, *p* < 0.001), and environmental (ρ = −0.449, *p* < 0.001) domains. In multivariable regression, IPSS remained independently associated with lower physical HRQoL (β = −0.768, *p* < 0.001), independent of age, socioeconomic status, and therapy type. *Conclusions*: Greater LUTS severity is associated with poorer health-related quality of life in men receiving pharmacological treatment for BPH. Integrating comprehensive symptom assessment with HRQoL measures may enhance clinical evaluation and support more personalized management. Longitudinal studies are needed to determine whether symptom improvement translates into meaningful gains in quality of life.

## 1. Introduction

Benign prostatic hyperplasia (BPH) is characterized by benign enlargement of the prostate gland resulting from uncontrolled hyperplastic proliferation of epithelial and fibromuscular tissues in the transition zone and periurethral region. It represents the predominant etiological cause of lower urinary tract symptoms (LUTS) in males. Men with BPH may exhibit symptoms such as decreased urine flow, increased frequency, hesitancy, post-void dribbling, and nocturia (voiding LUTS) [1,2]. Although symptom tolerance varies across individuals, LUTS in men with BPH are linked to broader health impacts beyond the urinary tract. Higher symptom burden is associated with poorer physical functioning, limitations in daily activities and self-care, greater pain/discomfort, and more anxiety/depressive symptoms, underscoring that LUTS affect both mental and physical health [3]. These effects can potentially lead to a decline in economic productivity and, in some cases, personal financial losses [4].

BPH is frequently undervalued and inadequately diagnosed. Most epidemiological studies have been conducted in regions with higher life expectancy, such as North America, Europe, and Australasia, while evidence from other settings remains limited [2]. Lifestyle and metabolic factors have been investigated as potential determinants of BPH, but findings remain inconsistent [5]. The International Prostate Symptom Score (IPSS), developed by the American Urological Association (AUA), is a validated, standardized patient-reported tool that quantifies the severity of LUTS related to BPH and assists in clinical evaluation, treatment planning, and research follow-up [6].

Management of moderate-to-severe LUTS includes pharmacological therapy, minimally invasive procedures, and surgery [7]. Monotherapy with α-blockers, 5-ARIs, anticholinergics, or PDE5 inhibitors is commonly initiated, while combination therapy with an α-blocker and a 5-ARI has shown greater efficacy in reducing symptoms and improving urinary flow [8].

Health-related quality of life (HRQoL) represents a multidimensional construct encompassing physical, psychological, and social aspects of functioning and perceived well-being. Over recent decades, HRQoL and overall quality of life (QoL) have gained increasing importance in medical research as complementary indicators to traditional biomedical outcomes. Despite this, the focus of many earlier studies remained limited to clinical or physiological parameters, often overlooking patients’ subjective experiences and well-being. Quality of life is now recognized as a key prognostic indicator and an essential component in evaluating treatment efficacy. It enables the identification of problems related to both physical and emotional dysfunction, thereby supporting more comprehensive, patient-centered care [9].

Socioeconomic factors play a crucial role in shaping HRQoL; however, their associations in older adults remain insufficiently established for the general elderly population. Investigating these correlations using contemporary data from aging cohorts could provide valuable new insights for public-health and quality-of-life research [10,11].

The growing global interest in assessing QoL prompted the World Health Organization (WHO) to develop a standardized conceptual framework through the WHO Quality of Life (WHOQOL) Group. The WHOQOL Group defined quality of life as “an individual’s perception of their position in life in the context of the culture and value systems in which they live and in relation to their goals, expectations, standards, and concerns” [12]. To operationalize this concept, the WHOQOL Group created the comprehensive WHOQOL-100, a 100-item scale assessing 24 dimensions of life quality [13]. However, due to its length and complexity, a shorter version, the WHOQOL-BREF, was subsequently developed. This abbreviated form retained two general questions on overall health and quality of life and one representative item from each of the 24 original domains [14]. The WHOQOL-BREF has since become one of the most widely used HRQoL instruments worldwide [15].

The substantial impact of BPH on patients’ quality of life, combined with its significant economic burden on healthcare systems, underscores the need for greater awareness and increased research investment beyond current levels [1]. Effective prevention and strategic management of BPH are essential to reduce its societal costs and safeguard the well-being of aging populations [16]. Accordingly, this cross-sectional study examined the association between LUTS severity (IPSS) and WHOQOL-BREF domain scores in men undergoing pharmacological treatment for BPH. A secondary objective was to evaluate whether age, socioeconomic status, and therapy type independently influence HRQoL outcomes.

## 2. Materials and Methods

### 2.1. Study Design and Ethics

This cross-sectional study was approved by the Ethics Committee of the University Clinical Centre of Vojvodina and conducted in accordance with the Declaration of Helsinki. Reporting adhered to STROBE recommendations for observational studies [17]. Written informed consent was obtained from all participants prior to enrolment.

### 2.2. Setting and Period

The study was conducted at the Clinic of Urology, University Clinical Centre of Vojvodina, from May to July 2024.

### 2.3. Participants and Eligibility Criteria

Inclusion criteria: (I) males aged 50–80 years; (II) provision of written informed consent; (III) pharmacotherapy for benign prostatic hyperplasia (BPH) for ≥12 months.

Exclusion criteria: refusal to consent; prior surgical treatment for BPH; any malignant disease; neurodegenerative disorders; or current use of medications known to affect LUTS/BPH symptoms (e.g., diuretics, antihistamines, calcium-channel blockers, phytotherapy, or tricyclic antidepressants).

**Figure 1:**
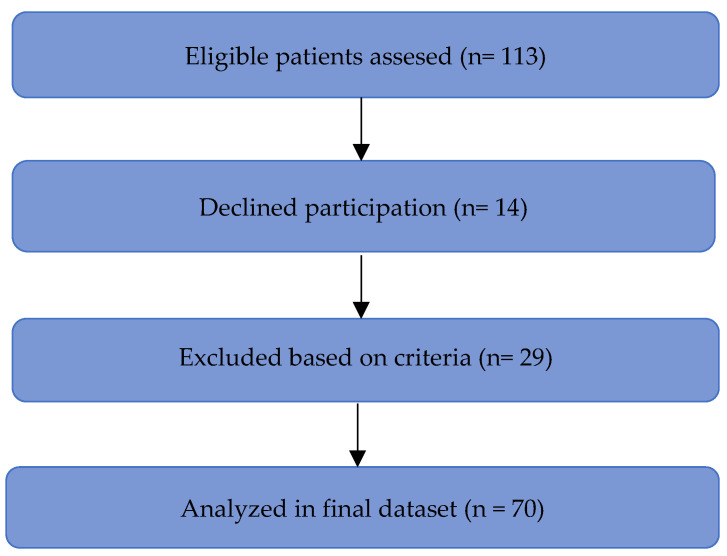
STROBE flow diagram of patient inclusion and analysis distribution.

Consecutive, eligible men attending scheduled outpatient urology consultations were invited. Of 113 eligible patients, 14 declined and 29 met exclusion criteria, yielding a final analytic sample of 70 participants (Figure 1).

### 2.4. Measures

#### 2.4.1. Lower Urinary Tract Symptoms

LUTS severity was assessed with the IPSS (7 items). Standard IPSS categories were applied: mild (0–7), moderate (8–19), and severe (20–35). The IPSS “bother” item (Q8: “If you were to spend the rest of your life with your urinary condition the way it is now, how would you feel about that?”) was rated on a 7-point scale from 0 (delighted) to 6 (terrible); higher scores indicate worse disease-related quality of life [6].

#### 2.4.2. Health-Related Quality of Life

HRQoL was measured using the WHOQOL-BREF, which comprises 24 items across four domains (physical, psychological, social relationships, and environment) and two general items on overall QoL and health. Items are scored on 5-point Likert scales (1–5). Domain raw scores were computed according to the WHOQOL-BREF manual and transformed from 4–20 to a 0–100 scale, with higher scores indicating better quality of life [18]. The Serbian-language version used in this study is a culturally validated translation. Item-level missingness was <1% across all items; therefore, a complete-case analysis approach was applied.

#### 2.4.3. Socioeconomic Status

Socioeconomic status (SES) was operationalized using the national benchmark for individual household consumption (in money and in kind) reported by the Statistical Office of the Republic of Serbia (87,973 RSD) and coded above vs. below the national average [19]. SES was dichotomized because the questionnaire captured income only as a binary category relative to the national average, without more granular socioeconomic indicators such as occupation or household economic status.

#### 2.4.4. Pharmacotherapy (Exposure)

Pharmacotherapy was categorized to improve clarity in the analysis. Monotherapy was defined as the use of a single pharmacological class only, including an α1-adrenergic blocker, a 5-α-reductase inhibitor (5-ARI), an anticholinergic agent, or a phosphodiesterase-5 inhibitor (PDE5I). Combination therapy was defined as the concurrent use of an α1-adrenergic blocker and a 5-ARI, consistent with guideline-recommended regimens for moderate-to-severe LUTS/BPH. Information on treatment duration, dosage, adherence, or previous therapy was not available. Because the monotherapy group comprises drug classes with different mechanisms of action, comparisons between monotherapy and combination therapy should be viewed as exploratory and descriptive rather than as assessments of relative therapeutic effectiveness. Evidence underpinning superior symptom reduction with combination regimens is noted in prior literature [8].

### 2.5. Data Collection Procedures

Questionnaires (IPSS and WHOQOL-BREF) were self-administered on site. Trained staff provided clarification upon request. Forms were reviewed at submission; missing items were completed immediately when possible; otherwise, complete-case analyses were performed.

### 2.6. Statistical Analysis

Continuous variables are presented as mean ± SD; categorical variables as counts (percentages). Between-group differences in WHOQOL-BREF domain scores across IPSS categories (mild/moderate/severe) were tested by one-way ANOVA with Tukey’s HSD post hoc comparisons. Assumptions of approximate normality and homogeneity of variance for ANOVA were evaluated using standard diagnostic procedures. The association between therapy type (monotherapy vs. combination) and IPSS category was examined using Pearson’s χ^2^ test. Associations between symptom burden and HRQoL were explored using Spearman’s rank correlations (IPSS total vs. each WHOQOL-BREF domain; IPSS bother item vs. domain scores). To evaluate independence of effects, multivariable linear regression models were fitted for each WHOQOL-BREF domain (outcomes), with age, SES (above vs. below national average), therapy type (monotherapy vs. combination), and IPSS included as covariates. Assumptions for linear regression were checked using standard diagnostic procedures. Variance inflation factors (VIFs) were examined to assess multicollinearity and indicated no concerning collinearity among included predictors. Residual diagnostics, including visual inspection of residual–versus–fitted plots, Q–Q plots, and Cook’s distance values, were used to evaluate normality, homoscedasticity, and influential observations. Two-tailed *p* < 0.05 was considered statistically significant. Given the exploratory nature of the study, no formal correction for multiple testing across WHOQOL-BREF domains was applied. Analyses were performed in IBM SPSS Statistics v26.0.

## 3. Results

### 3.1. Sociodemographic and Clinical Profile of Participants

The mean age of participants was 67.36 ± 8.66 years (range 50–80). Regarding education, most respondents had completed upper secondary (45.7%) or higher vocational (31.4%) programs, while a smaller proportion had attended vocational secondary (craft) schools or held a university degree. Only two participants reported primary education or less. The majority of patients were married (64.3%), whereas one quarter were single, and few were widowed or divorced. Monthly income was evenly distributed, with half of the sample reporting earnings above and half below the national average.

Most patients were receiving monotherapy (67.1%), while the remainder were treated with combination therapy (32.9%). According to IPSS classification, the majority of participants were moderately symptomatic (65.7%), followed by mild (20.0%) and severe (14.3%) categories. The association between therapy type (monotherapy vs. combination therapy) and IPSS symptom severity was not statistically significant, χ^2^ (2, N = 70) = 1.31, *p* = 0.52 (Table 1). These comparisons should be interpreted with caution given the heterogeneous composition of the monotherapy group and the absence of detailed data on treatment duration, dosage, and adherence. The distribution of patients across mild, moderate, and severe categories was broadly similar between therapy groups (Cramer’s V = 0.14, small effect size), with most classified as moderately symptomatic in both (70.2% vs. 56.5%, respectively).

### 3.2. HRQoL Differences Across LUTS Severity Levels

A one-way ANOVA was performed to compare WHOQOL-BREF domain scores across IPSS severity categories (Table 2). Statistically significant differences were observed in the Physical, Social Relationships, and Environmental domains, while the Psychological domain did not differ significantly between groups.

Post hoc Tukey analyses indicated that patients with severe LUTS reported significantly lower physical health scores compared with both mild (*p* = 0.002) and moderate (*p* = 0.046) groups. Similarly, social relationship scores were significantly lower in the severe group relative to both mild (*p* = 0.005) and moderate (*p* = 0.033) categories. In the environmental domain, quality-of-life scores were significantly lower for the severe group compared with moderate cases (*p* = 0.007), whereas the mild–moderate difference approached significance (*p* = 0.051). No statistically significant differences were detected in the psychological domain (*p* = 0.420). Effect sizes for between-group differences were in the small-to-moderate range across domains.

### 3.3. Correlation Between IPSSs and Quality of Life Domains

**Figure 2:**
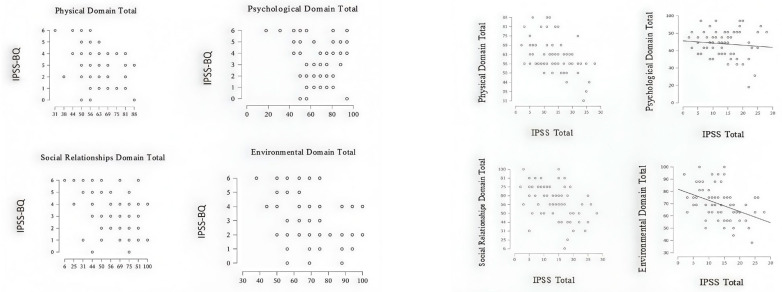
Correlation plots between IPSS measures and WHOQOL-BREF domains.

Spearman’s rank-order correlations between IPSS measures and WHOQOL-BREF domain scores are presented in Table 3 and illustrated in Figure 2. For the IPSS Bother Question (IPSS-BQ), weak to moderate negative correlations were observed with the Physical (ρ = −0.285, *p* = 0.017), Social (ρ = −0.341, *p* = 0.004), and Environmental (ρ = −0.315, *p* = 0.008) domains, whereas no significant relationship was found for the Psychological domain (ρ = 0.063, *p* = 0.606).

Similarly, the IPSS Total Score showed moderate negative correlations with the Physical (ρ = −0.438, *p* < 0.001), Social (ρ = −0.470, *p* < 0.001), and Environmental (ρ = −0.449, *p* < 0.001) domains, while no significant association was observed with the Psychological domain (ρ = −0.039, *p* = 0.751). The correlation plots in Figure 2 visually depict the distribution of WHOQOL-BREF domain scores across IPSS-BQ and IPSS Total measures. A denser clustering of points in the lower quality-of-life range is evident for the Physical, Social, and Environmental domains, consistent with the negative Spearman coefficients shown in Table 3, while no apparent pattern is observed for the Psychological domain.

### 3.4. Multivariable Predictors of Physical HRQoL

In the multivariable linear regression model (Table 4), the WHOQOL-BREF Physical domain score (0–100) was entered as the dependent variable. The full model showed acceptable fit (*R* = 0.563, *R*^2^ = 0.317, adjusted *R*^2^ = 0.188; *F*(11, 58) = 2.45, *p* = 0.014; RMSE = 10.29). Among all covariates, the IPSS total score remained the only statistically significant independent predictor of the physical domain score (*β* = −0.768, SE = 0.212, *p* < 0.001). Education variables also showed associations at or near the threshold of significance: upper secondary (*β* = −16.42, *p* = 0.043) and university degree (*β* = −18.22, *p* = 0.046) were significantly lower than the referent category, while vocational secondary and higher vocational college showed trend-level effects (*p* ≈ 0.09).

Age, therapy type, marital status, and income were not significant predictors (*p* > 0.05). After full adjustment for demographic and socioeconomic variables, IPSS remained the primary correlate, explaining approximately 32% of the variance in the physical HRQoL domain.

## 4. Discussion

This study found that higher LUTS severity was consistently associated with poorer HRQoL across multiple WHOQOL-BREF domains in men receiving pharmacological treatment for BPH. The strongest and most stable associations were observed for the physical, social, and environmental domains, whereas the psychological domain showed no clear pattern. These findings align with prior evidence linking LUTS burden to impairments in daily functioning and overall well-being.

In this single-centre cross-sectional study of men receiving pharmacological treatment for BPH, greater LUTS severity was consistently associated with lower HRQoL in the physical, social, and environmental domains of the WHOQOL-BREF, whereas no clear relationship was observed for the psychological domain. In the multivariable model, each one-point increase in IPSS corresponded to an approximately 0.77-point lower physical-domain score (β = −0.768, SE = 0.212, *p* < 0.001), with the model explaining ~32% of the variance (R^2^ = 0.317). Findings from the ANOVA were directionally consistent: patients with severe LUTS reported significantly lower physical, social, and environmental scores than those with milder categories, with no between-group differences for the psychological domain.

These domain-specific deficits are pathophysiologically plausible: nocturia and urgency disturb sleep and daily functioning, while frequency and leakage symptoms reduce mobility, promote activity avoidance, and contribute to social withdrawal. The need for constant toilet access and nocturnal rising further undermines perceived safety and environmental comfort, particularly among older adults [20]. Collectively, these findings reinforce that LUTS impose a broad systemic burden extending well beyond the urinary tract, impairing mobility, self-care, daily functioning, and overall well-being, with effects on physical and social domains comparable in magnitude to those seen in other chronic diseases [3,4,16].

In contrast to some reports suggesting that the single IPSS “bother” item correlates more strongly with HRQoL than the overall IPSS, our findings demonstrated the opposite pattern, with stronger and more consistent associations for the total IPSS score [21]. This likely reflects the greater reliability of a multi-item symptom index, which captures the cumulative impact and variability of LUTS more comprehensively and with less measurement error than a single global perception item [22]. The absence of a clear association for psychological domain likely reflects the limited sensitivity of a generic QoL instrument to mental-health constructs relevant to LUTS/BPH, combined with the modest sample size, which reduced power to detect smaller effects. Disease-specific mental-health measures such as validated scales for anxiety, depression, or sleep quality often reveal associations that generic instruments like the WHOQOL-BREF fail to capture, helping explain the heterogeneous findings reported across studies [23,24,25,26].

No significant differences in HRQoL or IPSS severity were observed between monotherapy and combination therapy in this sample. However, these findings should not be interpreted as evidence of comparable clinical effectiveness. The monotherapy category included several mechanistically distinct drug classes, while detailed information on treatment duration, dose intensity, prior therapy, and adherence was not available. Moreover, confounding by indication is unavoidable, as patients with more severe or persistent LUTS are more likely to receive combination therapy.

Taken together, these factors substantially limit the interpretability of between-group contrasts and underscore that therapy-related results are descriptive rather than inferential. Causal interpretation is not possible in a cross-sectional design, and longitudinal or interventional studies with detailed exposure measurement are needed to characterize domain-specific treatment effects more accurately [27,28,29,30].

The unexpected association between higher educational attainment and lower physical HRQoL in adjusted analyses should be interpreted cautiously. The reference education category comprised only two participants, raising concerns about sparse-data instability of dummy coefficients; moreover, SES was coarsely operationalized, as income was available only as a binary category without broader socioeconomic indicators. Residual confounding through occupational sedentariness, comorbidity burden or differing health expectations is plausible. Most prior studies report that higher education and income relate to better HRQoL in BPH, plausibly via greater health literacy, self-efficacy, and access to care [31]. Other work suggests income is more decisive: lower income independently predicts higher IPSS and more severe LUTS, while the effect of education weakens after accounting for income [32]. Future work should employ granular SES indicators and explicitly model behavioral/psychological mediators.

Routine integration of IPSS (including the “bother” item) with multidomain HRQoL assessment can identify patients at risk of functional impairment beyond urological symptoms and inform timely therapeutic adjustments. Where sensitivity and comparability are critical, combining WHOQOL-BREF with a BPH-specific measure (e.g., BPH Impact Index) and a generic utility scale (e.g., EQ-5D) may improve detection of meaningful change [33,34]. In clinical decision-making, evaluating observed changes against minimal clinically important difference (MCID) thresholds aids in assessing whether statistically significant changes result in patient-perceived benefits; recent research in a procedural context demonstrates the conceptualization of MCID concepts for LUTS outcomes [35]. However, no validated MCID thresholds exist for WHOQOL-BREF domains in LUTS/BPH populations, and therefore comparisons in the present study remain descriptive rather than anchored to clinically established benchmarks. Ultimately, employing standardized ICS nomenclature enhances comparability among studies and clinical communication, which is crucial for evaluating patient-reported outcomes in LUTS/BPH research [36].

The WHOQOL-BREF was an appropriate instrument for this study, offering a validated multidimensional approach suitable for older adults and enabling domain-specific interpretation of HRQoL [12,15,22].

Strengths of this research include the use of validated measures (IPSS, WHOQOL-BREF), prespecified analyses across domains, and multivariable adjustment. Limitations of this study include its cross-sectional design, which prevents assessment of temporality or causality, as well as its single-centre setting and modest sample size, particularly the small severe-LUTS subgroup, which limits precision and generalizability. Prospective calculations of minimum detectable difference were not performed, and retrospective estimation is not recommended. Residual confounding from unmeasured clinical variables remains likely, and effect estimates should be interpreted cautiously. Given the modest sample size, diagnostic checks have limited power to detect subtle deviations from model assumptions. Socioeconomic status was dichotomized due to restricted data availability, resulting in the loss of important gradient information. Incomplete information on comorbidities, BMI, psychological status, sleep quality, prostate volume, uroflowmetry, polypharmacy, and other clinically relevant variables may have introduced residual confounding. The categorization of pharmacotherapy is another limitation: monotherapy included several mechanistically different drug classes, while combination therapy referred only to concurrent use of an α-blocker and a 5-ARI, and no data were available on treatment duration, dosage, adherence, or prior therapy. For these reasons, therapy-group comparisons should be interpreted solely as exploratory. Finally, the use of a generic HRQoL instrument (WHOQOL-BREF) may not fully capture disease-specific impacts of LUTS/BPH, particularly in the psychological domain.

Future multicentre longitudinal studies incorporating detailed SES and comorbidity profiling, alongside disease-specific and generic HRQoL instruments, should determine whether reductions in IPSS translate into clinically meaningful domain-specific improvements under guideline-based care.

## 5. Conclusions

This cross-sectional study demonstrates that more severe LUTS are meaningfully associated with poorer health-related quality of life in men receiving pharmacological treatment for BPH. These findings highlight that the impact of LUTS extends beyond urinary symptoms and affects multiple aspects of daily functioning and well-being. The results reinforce the value of comprehensive symptom assessment in routine practice and underscore the limitations of generic HRQoL tools for capturing psychological burden. Future research should employ longitudinal designs, integrate disease-specific HRQoL instruments, and incorporate more detailed socioeconomic and clinical variables to clarify causal pathways and identify treatment strategies that yield clinically meaningful improvements in quality of life.

## Figures and Tables

**Table 1:** Baseline characteristics of the study participants (N = 70).

Characteristic	Category	N (%)
**Education**	Primary school or less	2 (2.86)
	Vocational secondary (craft school)	8 (11.43)
	Upper secondary (high-school diploma/matura)	32 (45.71)
	Higher vocational college (post-secondary)	22 (31.43)
	University degree (bachelor’s or higher)	6 (8.57)
**Marital status**	Single	18 (25.71)
	Married	45 (64.29)
	Divorced	1 (1.43)
	Widowed	6 (8.57)
**Income relative to national average**	Above average	35 (50.00)
	Below average	35 (50.00)
**Therapy**	Monotherapy	47 (67.14)
	Combination therapy	23 (32.86)
**IPSS categorization**	Mildly symptomatic	14 (20.00)
	Moderately symptomatic	46 (65.71)
	Severely symptomatic	10 (14.29)

**Table 2:** WHOQOL-BREF domain scores by IPSS severity (means ± SD) with one-way ANOVA and Tukey’s HSD test.

WHOQOL-BREF Domain	Mild Mean ± SD	Moderate Mean ± SD	Severe Mean ± SD	F (df = 2,67)	*p*	Post Hoc Summary (Tukey HSD)	95% CI (Lower-Upper)
**Physical**	68.43 ± 9.41	61.48 ± 10.49	52.50 ± 12.55	6.60	**0.002**	Severe < Mild (*p* = 0.002); Severe < Moderate (*p* = 0.046)	Moderate-Mild: −10.61–7.85 Severe-Mild: −20.26–4.77
**Psychological**	69.64 ± 12.00	68.26 ± 13.05	61.90 ± 25.47	0.88	0.420	n.s. (no significant group differences)	Mild: 62.72–76.57 Moderate: 64.42–72.33 Severe: 45.77–78.23
**Social Relationships**	68.71 ± 17.18	61.43 ± 16.81	46.90 ± 11.21	5.36	**0.007**	Severe < Mild (*p* = 0.005); Severe < Moderate (*p* = 0.033)	Moderate-Mild: −17.18–2.62; Severe-Mild: −35.24–−6.73
**Environmental**	77.86 ± 10.86	68.63 ± 13.42	61.40 ± 10.91	5.22	**0.008**	Severe < Moderate (*p* = 0.007); Mild–Moderate (*p* = 0.051)	Moderate-Mild: −16.93–−1.53; Severe-Mild: −26.90–−6.01

Note. WHOQOL-BREF = World Health Organization Quality of Life-BREF; IPSS = International Prostate Symptom Score. Bold *p* values indicate statistically significant results (*p* < 0.05).

**Table 3:** Spearman’s correlations between IPSS measures and WHOQOL-BREF domains.

A. IPSS Bother Question (IPSS-BQ)		
	**Spearman’s rho**	***p***
**Physical Domain**	−0.2850	**0.0168**
**Psychological Domain**	0.0627	0.6063
**Social Relationships Domain**	−0.3414	**0.0038**
**Environmental Domain**	−0.3145	**0.0080**
**B. IPSS Total Score**		
	**Spearman’s rho**	***p***
**Physical Domain**	−0.4380	**<0.001**
**Psychological Domain**	−0.0387	0.7505
**Social Relationships Domain**	−0.4702	**<0.001**
**Environmental Domain**	−0.4494	**<0.001**

Note. Bold *p* values indicate statistically significant correlations (*p* < 0.05). WHOQOL-BREF = World Health Organization Quality of Life-BREF; IPSS = International Prostate Symptom Score.

**Table 4:** Multivariable linear regression model for predictors of WHOQOL-BREF Physical domain score (0–100).

Variable	β (Unstandardized)	SE	Standardized β	t	*p*
**Intercept**	100.8100	13.7454	-	7.3341	**<0.001**
**IPSS total**	−0.7684	0.2121	−0.4133	−3.6230	**<0.001**
**Age**	−0.1724	0.1657	−0.1308	−1.0402	0.3026
**Combination therapy (vs. monotherapy)**	−3.2397	3.0082	-	−1.0769	0.2860
**Vocational secondary (craft school)**	−14.6376	8.5583	-	−1.7103	0.0925
**Upper secondary (high-school diploma/matura)**	−16.4180	7.9347	-	−2.0691	**0.0430**
**Higher vocational college (post-secondary, non-tertiary)**	−13.8846	7.9708	-	−1.7419	0.0868
**University degree (bachelor’s or higher)**	−18.2202	8.9177	-	−2.0432	**0.0456**
**Married (vs. single)**	0.2383	3.0661	-	0.0777	0.9383
**Divorced (vs. single)**	7.7926	11.1710	-	0.6976	0.4882
**Widowed (vs. single)**	−5.1873	5.1721	-	−1.0030	0.3201
**Income (less vs. more)**	−1.7150	2.6695	-	−0.6424	0.5231
Model summary: *R* = 0.563; *R*^2^ = 0.317; Adj *R*^2^ = 0.188; *F*(11, 58) = 2.45, ***p* = 0.014**; RMSE = 10.29.					

Note. Dependent variable: WHOQOL-BREF Physical domain (0–100). Bold *p* values indicate *p* < 0.05.

## Data Availability

The data sets and analysis presented in this study are available from the corresponding author after reasonable request.

## Abbreviations

The following abbreviations are used in this manuscript:

ANOVA	Analysis of Variance
AUA	American Urological Association
BPH	Benign Prostatic Hyperplasia
EQ-5D	EuroQol 5-Dimension Scale
HRQoL	Health-Related Quality of Life
ICS	International Continence Society
IPSS	International Prostate Symptom Score
IPSS-BQ	International Prostate Symptom Score–Bother Question
LUTSs	Lower Urinary Tract Symptoms
MCID	Minimal Clinically Important Difference
PDE5Is	Phosphodiesterase-5 Inhibitors
QoL	Quality of Life
SD	Standard Deviation
SE	Standard Error
SES	Socioeconomic Status
STROBE	Strengthening the Reporting of Observational Studies in Epidemiology
WHO	World Health Organization
WHOQOL	World Health Organization Quality of Life
WHOQOLBREF	World Health Organization Quality of Life–BREF version
